# Intracranial Squamous Cell Carcinoma Arising From a Cerebellopontine Angle Epidermoid Cyst Remnant Four Decades After Partial Resection

**DOI:** 10.3389/fonc.2019.00694

**Published:** 2019-07-30

**Authors:** Joshua A. Cuoco, Cara M. Rogers, Christopher M. Busch, Lisa S. Apfel, John J. Entwistle, Eric A. Marvin

**Affiliations:** ^1^Section of Neurosurgery, Carilion Clinic, Roanoke, VA, United States; ^2^Virginia Tech Carilion School of Medicine, Roanoke, VA, United States; ^3^Virginia Tech School of Neuroscience, Blacksburg, VA, United States; ^4^Edward Via College of Osteopathic Medicine, Blacksburg, VA, United States

**Keywords:** epidermoid cyst, squamous cell carcinoma, malignant transformation, malignant degeneration, cerebellopontine angle, brain neoplasm, neuro-oncology

## Abstract

Intracranial epidermoid cysts are benign lesions that typically remain asymptomatic; however, although histopathologically benign, these cysts can rarely undergo malignant transformation into squamous cell carcinoma. Primary intracranial squamous cell carcinoma carries a poor prognosis as optimal treatment modalities remain unclear due to their low incidence. Here, we present a case of a cerebellopontine angle epidermoid cyst remnant that underwent malignant transformation into squamous cell carcinoma 40 years after partial resection. To our knowledge, this case establishes the longest time interval to date for an intracranial epidermoid cyst to undergo malignant transformation. We also review the relevant literature and discuss recent retrospective clinical studies that have analyzed the effect of multimodal treatment approaches on survival outcomes in patients with these lesions.

## Background

Intracranial epidermoid cysts (ECs), also known as cholesteatomas, are rare benign, slow-growing lesions that account for 0.2–1.8% of intracranial tumors (1). Although classified as a benign entity based on histopathology and potentially curable with neurosurgical gross total resection, ECs can rarely undergo malignant transformation (MT) into squamous cell carcinoma (SCC). Since first described in 1912, only 15 cases of malignant cyst transformation have been reported to occur >5 years from time of initial surgery (2–16). Here, we describe a case of an EC remnant in the cerebellopontine angle (CPA) that underwent MT into SCC 40 years after partial resection. To our knowledge, this case establishes the longest time interval to date for an intracranial epidermoid cyst to undergo malignant transformation. The patient described in this report provided written informed consent for its publication.

## Case Presentation

**Figure 1:**
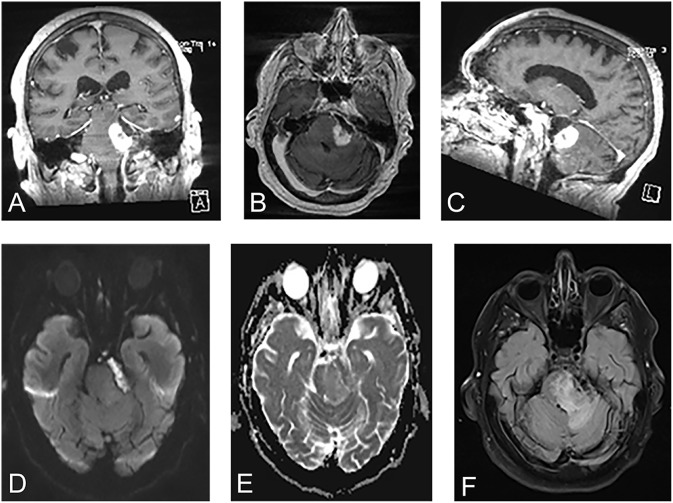
Pre-operative magnetic resonance images revealed a cystic lesion that was T1 hypointense, T2 hyperintense, and peripherally enhancing within the left cerebellopontine angle wrapping around the lateral brainstem. Adjacent to the cystic lesion was a T1 isointense, T2 hyperintense, avidly enhancing infiltrative component involving the brainstem, cerebellar peduncle, and cerebellum. **(A)** Coronal T1-weighted contrast enhanced. **(B)** Axial T1-weighted contrast enhanced. **(C)** Sagittal T1-weighted contrast enhanced. **(D)** Diffusion weighted-imaging. **(E)** Apparent diffusion coefficient. **(F)** Fluid-level attenuated inversion recovery.

A 71-year-old man presented to the emergency department with 2 months of progressively worsening diplopia, dizziness, headache, and ataxia. He also admitted to a 25-pound weight loss over the past few months. Past medical history was significant for partial resection of a left CPA EC 40 years prior and resection of urothelial carcinoma 2 years prior. Physical examination revealed a left abducens palsy, left facial droop, diminished hearing on the left as well as decreased sensation to light touch and pinprick on the left side of his face in all distributions of the trigeminal nerve. The cystic appearing component of the lesion was T1 hypointense, T2 hyperintense, and peripherally enhancing within the left CPA wrapping around the lateral brainstem. Adjacent to the cystic lesion was a T1 isointense, T2 hyperintense, avidly enhancing infiltrative component involving the brainstem, cerebellar peduncle, and cerebellum (Figure 1). A metastatic workup was negative. Initial differential diagnoses included: recurrent EC, SCC, arachnoid cyst, dermoid cyst, and neurenteric cyst. The patient was started on dexamethasone and scheduled for elective debulking of his brainstem lesion 2 weeks after initial hospitalization.

**Figure 2:**
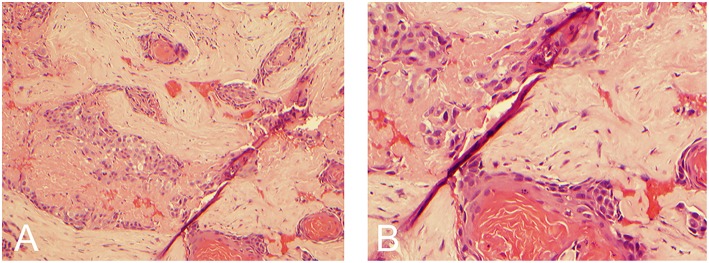
Hemotoxylin and eosin stain demonstrating keratin material with small clusters of atypical squamoid cells and keratin pearls consistent with squamous cell carcinoma. **(A)** 10x magnification. **(B)** 20x magnification.

Using the prior incision, the mass was approached with a standard retrosigmoid craniotomy. Stereotactic navigation and intraoperative neuromonitoring were utilized. Release of cerebrospinal fluid upon accessing the CPA afforded substantial relaxation of the cerebellum revealing a scarred capsule, which appeared similar to a pseudomeningocele. The capsule was then sharply incised and, once opened, stimulated with a monopolar probe to ensure cranial nerves were not in the vicinity. The enhancing portion of the lesion was extremely adherent to the brainstem. Furthermore, it was fibrous in nature and did not yield obvious planes for microsurgical dissection. The lesion was debulked from the inside out, expressing caseous material. The basilar artery, which was running through the capsule, was then skeletonized out. Pathology demonstrated keratinizing SCC with some sections consisting of almost entirely anucleated keratin material (Figure 2). Immunoreactivity for cytokeratin 5/6 was strong. Post-operatively, the patient regained sensation in the ophthalmic nerve dermatomal distribution; however, his other pre-operative symptomatology persisted. Adjuvant therapy included post-operative stereotactic radiosurgery with 25 Gy in five fractions.

## Discussion

Intracranial SCC is typically seen as a manifestation of metastases from a primary origin found outside the central nervous system or due to direct invasion of primary head and neck malignancy. However, although rare, MT of an intracranial EC into SCC is a well-documented phenomenon (2–23). According to Garcia et al. and Hamlat et al., true MT into SCC can only be classified as primary intracranial if it satisfies six criteria (Table 1) (17, 18). Moreover, as per the Hamlat et al. classification schema, MT of intracranial ECs can be sub-classified into five distinct entities including: (i) initial MT of an EC, (ii) MT from a remnant EC, (iii) MT with leptomeningeal carcinomatosis, (iv) SCC arising from other benign cysts, or (v) other malignancies arising from benign cysts (18). Since Ernst et al. reported the first case in 1912 there have been 74 documented cases of MT of an EC that have fulfilled the Garcia and Hamlat criteria (2–23). However, most of these previously reported cases were discovered within ECs at time of initial surgery or autopsy. Nevertheless, optimal treatment modalities as well as survival outcomes for these lesions remain unclear in the literature due their rarity.

**Table 1:** Criteria for diagnosis of malignant transformation of intracranial epidermoid cysts.

**Garcia's criteria**
1. Restriction to intracranial, intradural compartment
2. No invasion or extension beyond the dura, cranial bones, or intracranial orifices
3. No communication with middle ear, air sinuses, or sella turcica
4. No evidence of nasopharyngeal tumor
**Hamlat's additional criteria**
5. Presence of a benign squamous cell epithelium within the malignant tumor
6. Exclusion of metastatic carcinoma

Our literature review found that it is extremely rare for MT of an EC to occur >5 years from initial surgery. Indeed, only 15 cases (excluding the present case) have been reported to date (Table 2) (2–16). Of the reported cases, age at diagnosis of malignant cyst transformation ranged between 37 and 74 years with an average age of 54 years. Of these 15 cases, 10 patients (66.7%) were females. All 15 patients underwent surgical resection. Seven patients were subsequently treated with adjuvant radiotherapy or stereotactic radiosurgery. The interval from first surgical intervention to MT ranged from 5 to 33 years with a mean of 15.4 years. The most common location of the lesion was the CPA. Here, we presented a case of a 71 year-old male who was originally diagnosed with a CPA benign EC at the age of 31. Remarkably, after 40 years of clinical silence, his EC remnant underwent MT into SCC.

**Table 2:** Summary of reported cases of malignant transformation of intracranial epidermoid cysts occurring >5 years from original diagnosis.

**Year**	**Author**	**Age/Sex**	**Location**	**Treatment**	**Interval from EC**	**Clinical outcome**	**References**
1965	Fox and South	43M	Temporal	Sx	7 years	Died 1 month	(2)
1987	Goldman and Gandy	59F	Lateral Ventricle	Sx + Rx	33 years	Alive 3 years	(3)
1989	Abramson et al.	37M	CPA	Sx	5 years	N/A	(4)
1991	Tognetti et al.	67F	Frontotemporal	Sx	31 years	Died 1 month	(5)
1994	Radhakrishnan et al.	53M	Frontal	Sx + Rx	31 years	Alive 1 month	(6)
1999	Murase et al.	50F	CPA	Sx + SRS + Ch	10 years	Alive 5 years	(7)
2001	Asahi et al.	55F	CPA	Sx	13 years	Died 3 months	(8)
2004	Guan et al.	42F	Temporal	Sx + Rx	17 years	Alive 1 year	(9)
2006	Tamura et al.	56F	CPA	Sx + SRS	8 years	Alive 13 months	(10)
2009	Ge et al.	50M	Temporal	Sx	6 years	N/A	(11)
2010	Hao et al.	61F	CPA	Sx	6 years	Died 1 month	(12)
2010	Kano et al.	64F	CPA	Sx + Rx	16 years	Died 25 months	(13)
2010	Nakao et al.	74F	CPA	Sx + Rx	20 years	Alive 17 months	(14)
2017	Ozutemiz et al.	64M	Lateral Ventricle	Sx	23 years	Rec 3 months	(15)
2017	Mascarenhas et al.	35F	CPA	Sx	5 years	N/A	(16)
2019	Cuoco et al.	71M	CPA	Sx + SRS	40 years	Alive 4 months	

*EC, epidermoid cyst; M, male; F, female; Sx, surgery; Rx, radiation; CPA, cerebellopontine angle; SRS, stereotactic radiosurgery; Ch, chemotherapy; Rec, recurrent; N/A, not available*.

Although surgical resection is the current standard of care for the treatment of intracranial ECs, there is currently no accepted treatment modality for cysts that malignantly transform. Several retrospective clinical studies have attempted to define optimal treatment modalities. Hamlat et al. determined the mean survival in patients who underwent surgical resection (*n* = 9) vs. surgical resection plus post-operative radiotherapy ± chemotherapy (*n* = 24) to be 10 and 28 months, respectively (log-rank test, *P* = 0.077) (18). Although statistically non-significant, the authors advocated for adjuvant radiotherapy as most patients in their cohort who underwent this adjuvant treatment survived for >1 year. Similarly, Nagasawa et al. reviewed overall survival data in 36 patients who underwent surgical resection vs. surgical resection plus post-operative radiotherapy (20). They found that compared to a mean survival of 6.6 months in patients treated with surgery alone (*n* = 18), patients treated with surgery plus adjuvant radiotherapy (*n* = 18) demonstrated an increase in mean survival to 12.7 months (log-rank test, *P* < 0.003) (20). Importantly, no significant correlation was noted between dose of radiation and survival outcome. Tamura et al. performed a meta-analysis for survival outcomes in 24 cases investigating the clinical benefit of post-operative stereotactic radiosurgery (10). The authors found the median survival times for the surgery alone group (*n* = 9), surgery with radiotherapy group (*n* = 11), and surgery with stereotactic radiosurgery group (*n* = 4) to be 1, 18, and 44 months, respectively (log-rank test, *P* < 0.004) (10). Although limited by sample size and publication bias, these prior two studies retrospectively found a statistically significant short-term survival benefit with use of surgery with adjuvant radiotherapy or adjuvant stereotactic radiosurgery compared to surgery alone.

Further clinical studies have investigated the potential benefit of a multimodal treatment approach compared to surgery alone or surgery plus post-operative radiotherapy. In a subsequent report by Nagasawa et al., the authors reviewed 58 cases and found a mean survival time of 5.3 months with palliative management (*n* = 11), 25.7 months with chemotherapy either alone or in combination with surgery or other adjuvant therapies (*n* = 6), 29.2 months with either stereotactic radiosurgery alone or in combination with surgery or other adjuvant therapies (*n* = 5), and 36.3 months with surgery plus two or more adjuvant therapies (*n* = 4) (21). Survival outcomes for the stereotactic radiosurgery group ± surgery or other adjuvant therapies, chemotherapy group ± surgery or other adjuvant therapies, and surgery plus two or more adjuvant modalities group were found to be statistically significant compared to the surgery alone group (21). Liu et al. reviewed 72 cases and found that the mean survival of surgery alone (*n* = 25) vs. surgery with post-operative radiotherapy (*n* = 25) to be 6.9 and 11.9 months, respectively (22). Furthermore, the authors found a mean survival of surgery and stereotactic radiosurgery (*n* = 3), chemotherapy alone or in combination with other treatments (*n* = 3), and surgery and two or more adjuvant treatments (*n* = 4) to be 23.3, 13.7, and 36.5 months, respectively (22). However, the authors emphasize that the only statistically significant difference in mean survival outcomes was between the surgery with post-operative radiotherapy group and surgery with stereotactic radiosurgery group (*P* < 0.05) (22). Kwon et al. determined the median survival for patients treated with surgery alone (*n* = 24) vs. surgery with radiotherapy or stereotactic radiosurgery (*n* = 30) to be 5 and 35 months, respectively (*P* = 0.037) (23). Patients who underwent gross total resection (*n* = 7) vs. subtotal resection (*n* = 35) demonstrated a difference in median survival of 48 and 25 months, respectively (*P* = 0.067) (23). Moreover, in the same study, the median survival for patients who underwent surgery plus radiotherapy or stereotactic radiosurgery with chemotherapy (*n* = 5) vs. without chemotherapy (*n* = 25) was found to be 35 and 12 months, respectively (*P* = 0.676) (23). Although unable to provide a definitive standard of treatment, these retrospective clinical studies described in prior emphasize that a multimodal treatment approach may yield the best survival outcomes for patients with intracranial SCC.

Here, we present a rare case of primary SCC in the CPA treated with surgical resection followed by post-operative stereotactic radiosurgery. Of the 15 reported cases of malignant cyst transformation occurring >5 years from time of initial surgery, only two cases incorporated post-operative stereotactic radiosurgery in their treatment regimen (7, 10). We decided to pursue surgical resection followed by post-operative radiotherapy as the current literature (described in prior) reports significantly improved survival outcomes with this regimen compared to surgery alone (10, 20, 21, 23). Moreover, we specifically advocated for stereotactic radiosurgery, rather than traditional external beam radiotherapy, based upon favorable survival outcomes in the described literature with stereotactic radiosurgery (10, 21, 22). Although several retrospective studies have demonstrated clinical benefit with post-operative radiotherapy as well as a multimodal treatment approach, prospective trials are obligatory to define a standard of care for primary intracranial SCC.

## Conclusions

Malignant transformation of an intracranial EC is a rare occurrence with strict criteria defining its diagnosis. Here, we described a case of an EC remnant in the CPA which underwent MT into SCC 40 years after partial resection. To our knowledge, this case establishes the longest time interval to date for an intracranial EC to undergo MT.

## Data Availability

The raw data supporting the conclusions of this manuscript will be made available by the authors, without undue reservation, to any qualified researcher.

## Ethics Statement

The patient described in this report provided written informed consent for its publication.

## Author Contributions

JC: Primary author of manuscript. JC, CR, CB, LA, JE, and EM: Provided substantial contributions to the conception and design of the manuscript, contributed to manuscript revision, read, and approved the submitted version and agree to be accountable for all aspects of the work ensuring that questions related to the accuracy or integrity of any part of the work are investigated and resolved.

### Conflict of Interest Statement

The authors declare that the research was conducted in the absence of any commercial or financial relationships that could be construed as a potential conflict of interest.

## Abbreviations

EC: epidermoid cyst
MT: malignant transformation
SCC: squamous cell carcinoma
CPA: cerebellopontine angle.

## References

[1] Busch CM Prickett JT Stein R Cuoco JA Marvin EA Witcher MR Meckel's cave epidermoid cyst presenting as multiple cranial nerve deficits due to indirect tumoral compression of the cavernous sinus: a case report and literature review. World Neurosurg. (2019) 121:88–94. 10.1016/j.wneu.2018.09.206 30308341

[2] Fox H South EA. Squamous cell carcinoma developing in an intracranial epidermoid cyst (cholesteatoma). J Neurol Neurosurg Psyciatry. (1965) 28:276–81. 10.1136/jnnp.28.3.276 14345685 PMC495902

[3] Goldman SA Gandy SE. Squamous cell carcinoma as a late complication of intracerebroventricular epidermoid cyst. Case Report. J Neurosurg. (1987) 66:618–20. 10.3171/jns.1987.66.4.0618 3559730

[4] Abramson RC Morawetz RB Schlitt M. Multiple complications from an intracranial epidermoid cyst: case report and literature review. Neurosurgery. (1989) 24:574–8. 10.1097/00006123-198904000-00014 2651960

[5] Tognetti F Lanzino G Manetto V Calbucci F. Intracranial squamous cell carcinoma arising in remnant of extirpated epidermoid cyst. Br J Neurosurg. (1991) 5:303–5. 10.3109/02688699109005191 1892574

[6] Radhakrishnan VV Saraswathy A Rout D. Squamous-cell carcinoma arising in the remnant of an extirpated interacerebral epidermoid cyst. Indian J Pathol Microbiol. (1994) 37:S27–8. 8613163

[7] Murase S Yamakawa H Ohkuma A Sumi Y Kajiwara M Takami T. Primary intracranial squamous cell carcinoma–case report. Neurol Med Chir. (1999) 39:49–54. 10.2176/nmc.39.49 10093462

[8] Asahi T Kurimoto M Endo S Monma F Ohi M Takami M. Malignant transformation of cerebello-pontine angle epidermoid. J Clin Neurosci. (2001) 8:572–4. 10.1054/jocn.2000.0856 11683611

[9] Guan LM Qi XX Zhang JR Xu K Cui LJ Zhang Q. Intracranial squamous cell carcinoma developing in remnant of an epidermoid cyst: case report and literature review. Chin Med J. (2004) 117:1880–3. 15603727

[10] Tamura K Aoyagi M Wakimoto H Tamaki M Yamamoto K Yamamoto M. Malignant transformation eight years after removal of a benign epidermoid cyst: a case report. J Neurooncol. (2006) 79:67–72. 10.1007/s11060-005-9117-6 16583265

[11] Ge P Luo Y Fu S Ling F. Recurrent epidermoid cyst with malignant transformation into squamous cell carcinoma. Neurol Med Chir. (2009) 49:442–4. 10.2176/nmc.49.442 19779295

[12] Hao S Tang J, Wu Z, Zhang L, Zhang J, Wang Z. Natural malignant transformation of an intracranial epidermoid cyst. J Formos Med Assoc. (2010) 109:390–6. 10.1016/S0929-6646(10)60068-X 20497873

[13] Kano T Ikota H Kobayashi S Iwasa S Kurosaki S Wada H. Malignant transformation of an intracranial large epidermoid cyst with leptomeningeal carcinomatosis: case report. Neurol Med Chir. (2010) 50:349–53. 10.2176/nmc.50.349 20448435

[14] Nakao Y Nonaka S Yamamoto T Oyama K Esaki T Tange Y. Malignant transformation 20 years after partial removal of intracranial epidermoid cyst–case report. Neurol Med Chir. (2010) 50:239–9. 10.2176/nmc.50.236 20339276

[15] Ozutemiz C Ada E Ersen A Ozer E. Imaging findings of an epidermoid cyst with malignant transformation to squamous cell carcinoma. Turk Neurosurg. (2017) 27:312–5. 10.5137/1019-5149.JTN.12722-14.0 27349393

[16] Mascarenhas A Parsons A Smith C Molloy C Jukes A. Malignant squamous cell carcinoma arising in a previously resected cerebellopontine angle epidermoid. Surg Neurol Int. (2017) 8:186. 10.4103/sni.sni_99_17 28868198 PMC5569395

[17] Garcia CA McGarry PA Rodriguez F. Primary intracranial squamous cell carcinoma of the right cerebellopontine angle. J Neurosurg. (1981) 54:824–8. 10.3171/jns.1981.54.6.0824 7017078

[18] Hamlat A Hua ZF Saikali S Laurent JF Gedouin D Ben-Hassel M Guegan Y Malignant transformation of Intracranial epithelial cysts: systematic article review. J Neurooncol. (2005) 74:187–94. 10.1007/s11060-004-5175-4 16193391

[19] Ernst P Haufung dysontogenetischer Bildungen am Zentral-nervensystems. Verhandl Dtsch Path Gesellsch. (1912) 15:226–30.

[20] Nagasawa D Yew A Spasic M Choy W Gopen Q Yang I. Survival outcomes for radiotherapy treatment of epidermoid tumors with malignant transformation. J Clin Neurosci. (2012) 19:21–6. 10.1016/j.jocn.2011.06.002 22024232

[21] Nagasawa DT Choy W Spasic M Yew A Trang A Garcia HM. An analysis of intracranial epidermoid tumors with malignant tumors: treatment and outcomes. Clin Neurol Neurosurg. (2013) 115:1071–8. 10.1016/j.clineuro.2012.10.026 23219403

[22] Liu X Chen Z Dong Y He X Pan X Tong D. Primary intracranial squamous cell carcinoma arising de novo: a case report and review of the literature. World Neurosurg. (2018) 120:372–81. 10.1016/j.wneu.2018.08.067 30144612

[23] Kwon SM Kim JH Kim YH Hong SH Kim CJ Treatment and survival outcomes of primary intracranial squamous cell carcinoma. World Neurosurg. (2018) 125:1–9. 10.1016/j.wneu.2018.11.252 30576830

